# Fabrication and Supercritical Water Corrosion Resistance of TiN/TiNC/Al_2_O_3_ Multilayer Coating on Inconel 625 Alloy by CVD Method

**DOI:** 10.3390/ma15217670

**Published:** 2022-10-31

**Authors:** Zhidong Zhang, Yuelong Pan, Hao Guo, Xiangcai Zeng, Qiwu Shi, Tiecheng Lu

**Affiliations:** 1 China Nuclear Power Engineering Co., Ltd., Shenzhen 518124, China; 2 College of Physics, Sichuan University, Chengdu 610064, China; 3 ET Diamond Technology, Chengdu 610064, China; 4 College of Materials Science and Engineering, Sichuan University, Chengdu 610064, China

**Keywords:** multilayer coating, Inconel 625 alloy, chemical vapor deposition, supercritical water corrosion

## Abstract

A TiN/TiNC/Al_2_O_3_ multilayer coating was deposited on an Inconel 625 alloy by the chemical vapor deposition method as a protective barrier to improve the corrosion resistance in supercritical water. The corrosion characteristics were evaluated in a reactor at 500 °C and 25 MPa for 72 h. The surface morphology of the coated samples was relatively dense with no obvious cracks or pores observed. The XRD analysis revealed that the coatings were composed of TiN, TiNC and α-Al_2_O_3_ phases. After exposure to supercritical water, the surface morphology of the coatings was still dense and kept integrity. The phase composition of the coatings was also not changed, with no obvious corrosion scales detected. This result demonstrates the effectiveness of TiN/TiNC/Al_2_O_3_ coatings as a protective coating under harsh supercritical water environments.

## 1. Introduction

Supercritical water (SCW) is defined as the water beyond the critical thermodynamic point (374 °C and 22.1 MPa), which has unique physical and chemical properties, such as low viscosity and excellent heat conductivity and diffusivity properties [1,2]. In addition, the solubility of the organic substances and gases is increased significantly in supercritical water. Supercritical water oxidation (SCWO) is a promising wastewater treatment technology, showing high destruction efficiency for a broad range of organic wastes and only producing harmless end products [3]. During the SCWO process, oxidizing agents and organic substances are fully dissolved in supercritical water, and the homogeneous oxidation reaction is carried out rapidly. In a few minutes, organic substances are thoroughly transformed into harmless compounds in the form of small molecules, such as N_2_, CO_2_ and H_2_O. Compared with other advanced oxidation technologies for the treatment of high concentration organic waste, such as wet-air oxidation and incineration, SCWO technology has many outstanding advantages [4]. For example, the removal efficiency of organic matter after a SCWO process can reach up to 99.9% within just a few minutes, and self-heating balance can be reached when the proportion of organic material is more than 3 wt.%. However, the supercritical water environment involves elevated temperature and pressure with strong oxidation, which enhances both chemical and electrochemical corrosion of structural reactor materials [5,6,7,8]. One promising approach to improve the corrosion resistance of structural reactor materials is to form a corrosion-resistant coating material on their surface [9,10].

In nuclear applications, titanium nitride (TiN) is widely used as a protective coating material in various industries for its desirable mechanical and chemical properties, including superior wear resistance, high hardness, excellent corrosion resistance and low friction coefficient [11,12,13,14]. Studies by Khatkhatay et al. found that the TiN coating formed on the polished zircaloy-4 substrate can gain significant enhancement of corrosion resistance in supercritical environments [15]. TiNC is also a reliable hard coating material with outstanding mechanical and tribological properties, including ultra-high hardness, high corrosion resistance, high thermodynamic stability and comparatively good toughness. In contrast to TiN, TiNC coatings predominate with their better anti-adhesive and anti-abrasive capabilities. Furthermore, alumina-based oxide scales are known to mitigate many types of corrosion found in SCW conditions such as stress corrosion cracking, pitting and general corrosion [16]. It is known that double layer coatings often have a better performance than single layer coatings in terms of corrosion and oxidation resistance [17]. Thus, it is of great interest to develop a TiN/TiNC/Al_2_O_3_ multilayer coating to improve the corrosion resistance of reactor materials in supercritical water.

In this paper, we prepared a TiN/TiNC/Al_2_O_3_ multilayer coating on Inconel 625 Ni-based superalloy via a chemical vapor deposition process. The morphology and phase composition of the coated samples before and after the supercritical water oxidation test were characterized by means of XRD, SEM and EDS analyses. The supercritical water oxidation test was performed at 500 °C and 25 Mpa for 72 h in a batch reactor. The pH was adjusted to 5 using CO_2_ and the oxygen concentration was 1000 mg/L.

## 2. Experimental Section

### 2.1. Materials and Sample Preparation

The substrate material used in this study was an Inconel 625 nickel base alloy with a sample size of Φ15 mm × 2 mm. The samples were polished with metallographic sandpaper and then ultrasonically cleaned by a metal cleaning agent and dried. The TiN/TiNC/Al_2_O_3_ coatings were formed on Inconel 625 alloys in the CVD equipment SCT-600H manufactured by the SurcoTec company. The deposition process for TiN, TiNC and Al_2_O_3_ was conducted for 1 h, 2 h and 10 h at a temperature of 1010 °C, respectively. The reaction equations for the deposition process are shown as follows:

4H_2_ + N_2_ + 2TiCl_4_ → 2TiN + 8HCl

N_2_ + 2TiCl_4_ + 2CH_4_ → 2TiNC + 8HCl

2Al + 6HCl → 2AlCl_3_ + 3H_2_

2AlCl_3_ + 3CO_2_ + 3H_2_ → Al_2_O_3_ + 6HCl + 3CO

### 2.2. Characterization

Phases of the coatings were identified by an X-ray diffractometer (DX-2700). The macroscopic morphology of the coated samples was observed using a digital camera. Scanning electron microscopy coupled with energy dispersive X-ray spectrometry (JSM-IT500HR) was used to investigate the surface morphology and cross-sectional composition of the specimens before and after supercritical water oxidation. The deionized water and a certain amount of hydrogen peroxide were added into the reactor, and the oxygen concentration was calculated by the formula 2H_2_O_2_ → 2H_2_O + O_2_.

## 3. Results

### 3.1. Characterization of the TiN/TiNC/Al_2_O_3_ Coating

**Figure 1:**
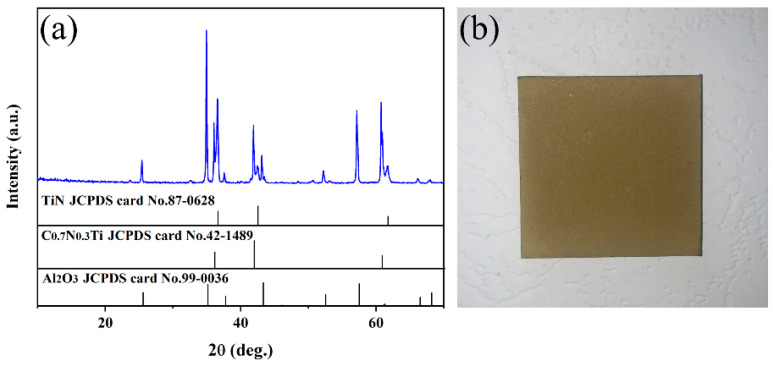
(**a**) Surface XRD pattern of the TiN/TiNC/Al_2_O_3_ coating. (**b**) Real object diagram of the coating prepared on surface of Inconel 625 alloy.

The XRD patterns of the multilayer TiN/TiNC/Al_2_O_3_ coating deposited by the CVD method are presented in Figure 1a. It can be clearly observed that the coatings are mainly composed of TiN (JCPDS: 97-0628), TiNC (JCPDS: 42-1489) and α-Al_2_O_3_ phases (JCPDS: 99-0036), with no other impurity phases detected. It is indicated that the multilayer coatings exhibit a favorable phase purity. Figure 1b presents the digital photo of the coated Inconel 625 samples. It can be seen that the surface of the coated samples exhibits a light yellow color, and it is smooth and compact with no obvious macroscopic defects observed. It is indicated that the CVD process is a satisfactory technique for the fabrication of fine multilayer ceramic coatings.

**Figure 2:**
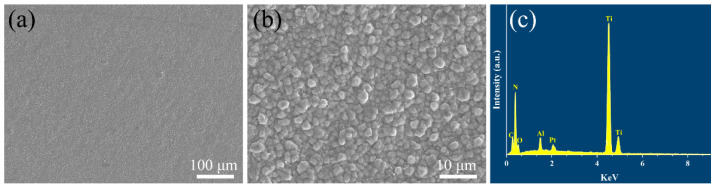
(**a**,**b**) Surface microstructure and (**c**) corresponding energy dispersive X-ray spectrum of TiN/TiNC/Al_2_O_3_ coating.

Figure 2 exhibits the surface morphology and corresponding EDS element analysis of the coatings deposited by the CVD method. It is clearly observed that the coatings have a homogeneous and dense structure with no cracks or holes seen on the surface of the coatings. From the high magnification of the SEM image in Figure 2b, it can be seen that the ceramic grains on the coating surface are bonded with each other tightly and the grain size has a diameter of about 2 μm.

**Figure 3:**
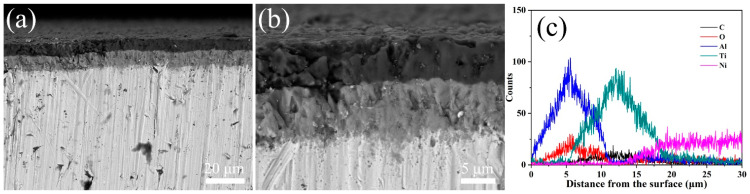
(**a**,**b**) Cross-sectional morphology of TiN/TiNC/Al_2_O_3_ coatings and (**c**) elemental distribution by EDS.

The performance of the protective coating is significantly influenced by the coating thickness. The coated samples were cut and polished by a 2000 mesh sandpaper for the characterization of the cross-section morphology. Figure 3 shows the cross-sectional backscattered electron (BSE) images of the coated Inconel 625 samples and the elemental distribution along the coating by EDS analysis. It can be seen that the coatings have a two-layer structure with a total thickness of 20 μm. The outer and inner layer in the coatings are the Al_2_O_3_ and TiNC layers, respectively. Both of the thicknesses of the two layers are about 10μm. The two different coating layers and the Inconel 625 substrate are in a good combination with each other, as no gap can be observed between the coatings and the substrate.

### 3.2. Characterization after Supercritical Water Oxidation Test without Oxygen

**Figure 4:**
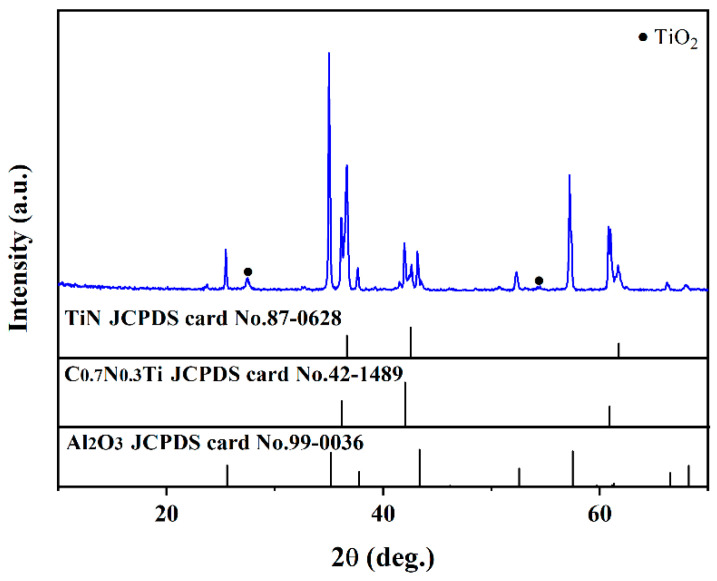
Surface XRD pattern of the TiN/TiNC/Al_2_O_3_ coatings after supercritical water test (500 °C, 25 Mpa, 72 h).

Figure 4 shows the XRD patterns of the multi-layer TiN/TiNC/Al_2_O_3_ coatings after the supercritical water corrosion test for 72 h (500 °C, 25 Mpa, 72 h). It can be clearly observed that the main components of the coating were still TiN, TiNC and Al_2_O_3_ phases. There is a weak peak detected at about 27.5°, which is difficult to be recognized.

**Figure 5:**
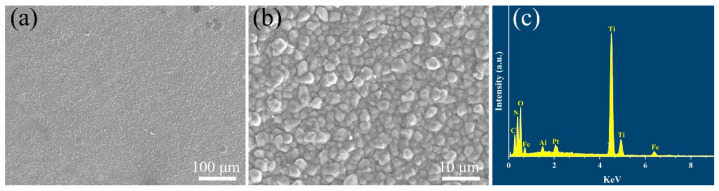
(**a**,**b**) Surface microstructure and (**c**) energy dispersive X-ray spectrum of the TiN/TiNC/Al_2_O_3_ coatings after SCWO test (500 °C, 25 Mpa, 72 h).

The surface morphology and element composition of the coatings after the supercritical water oxidation test via SEM and EDS analyses are shown in Figure 5. It can be seen that the surface of the coatings still has a uniform and dense microstructure with no changes compared with that before the supercritical water oxidation test. The element compositions are also not changed except for the presence of the Fe element, which may be due to the samples being polluted by other specimens in the supercritical water reactor. It can be concluded that the as-prepared multi-layer TiN/TiNC/Al_2_O_3_ ceramic coatings have a satisfactory corrosion resistance under a harsh supercritical water oxidation environment.

### 3.3. Characterization after Supercritical Water Oxidation Test with Oxygen

**Figure 6:**
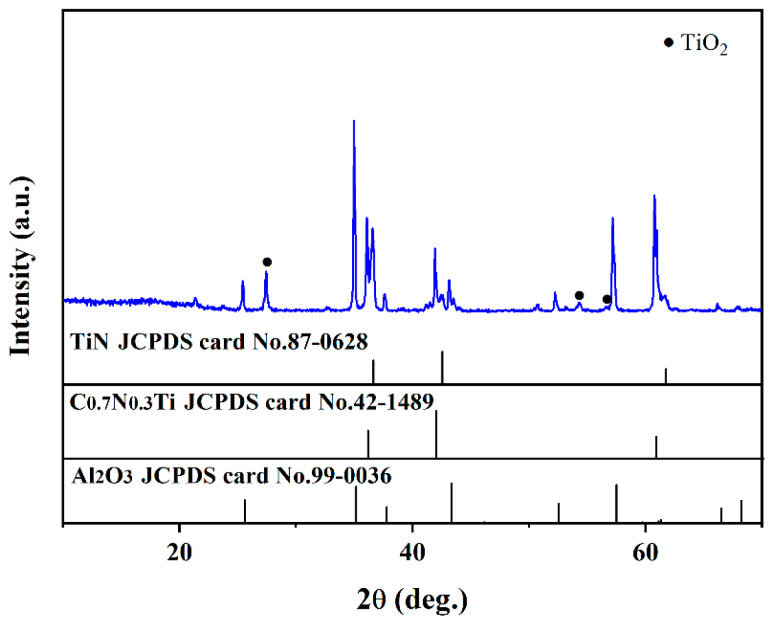
Surface XRD pattern of the TiN/TiNC/Al_2_O_3_ coatings after supercritical water test (500 °C, 25 Mpa, 72 h, PH = 5, oxygen concentration = 1000 mg/L).

XRD patterns of the coatings after the supercritical water oxidation test are shown in Figure 6 (500 °C, 25 Mpa, 72 h, PH = 5, oxygen concentration = 1000 mg/L). It can be seen that the composition has no evident change after the supercritical water oxidation test. The element compositions are also not changed except for the presence of the Fe element, which may be due to the samples were polluted by other specimen in the supercritical water reactor. It can be concluded that the as-prepared multi-layer TiN/TiNC/Al_2_O_3_ ceramic coatings have a satisfied corrosion resistance under a harsh supercritical water oxidation environment.

**Figure 7:**
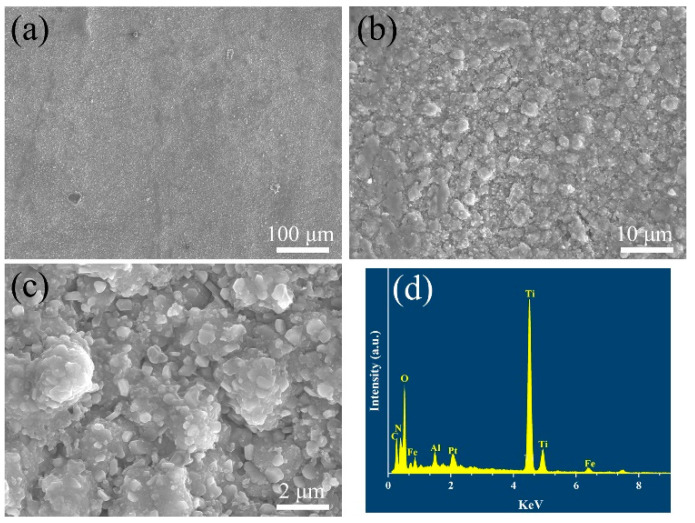
(**a**–**c**) Surface microstructure and (**d**) energy dispersive X-ray spectrum of the TiN/TiNC/Al_2_O_3_ coatings after SCWO test (500 °C, 25 Mpa, 72 h, pH = 5, oxygen concentration = 1000 mg/L).

Figure 7 illustrates the coating microstructures and compositions after the supercritical water oxidation test. It can be seen that there are also no changes in the cross-sectional morphology after the supercritical water oxidation test. No holes or cracks in the coatings can be found, indicating the multilayer ceramic coatings could effectively inhibit the oxidation of supercritical water.

## 4. Conclusions

The TiN/TiNC/Al_2_O_3_ composite coatings with thicknesses of 20 μm were fabricated on an Inconel 625 alloy by the CVD method, and their corrosion behaviors were evaluated in a batch reactor at 500 °C and 25 MPa for 72 h. The as-deposited coatings exhibit high phase purity, and the surface morphology is relatively dense and homogeneous with no cracks or pores observed. The coatings exhibit a two-layer structure composed of the outer Al_2_O_3_ layer and the inner TiNC layer. Both the Al_2_O_3_ layer and TiNC layer have a thickness of 10 μm. The supercritical water oxidation test shows that the as-deposited multilayer coatings exhibit an excellent corrosion resistance property under a supercritical water environment, with no changes in surface morphology and phase composition of the coatings after the oxidation test.
